# Evaluation of Pre-harvest Microbiological Safety of Blueberry Production With or Without Manure-Derived Fertilizer

**DOI:** 10.3389/fmicb.2019.03130

**Published:** 2020-01-14

**Authors:** Xiaoye Shen, Lina Sheng, Chris Benedict, Chad E. Kruger, Yuan Su, Elizabeth Schacht, Yifan Zhang, Mei-Jun Zhu

**Affiliations:** ^1^School of Food Science, Washington State University, Pullman, WA, United States; ^2^Whatcom County Extension, Washington State University, Bellingham, WA, United States; ^3^Center for Sustaining Agriculture and Natural Resources, Washington State University, Pullman, WA, United States; ^4^Department of Nutrition and Food Science, Wayne State University, Detroit, MI, United States

**Keywords:** manure-derived ammonium sulfate, blueberry, coliform, Shiga toxin-producing *Escherichia coli*, *Salmonella*, *Listeria monocytogenes*

## Abstract

Blueberry is an important commodity in Washington State, which was one of the leading blueberry producers in the United States. As a ready-to-eat fruit, blueberry has no or limited post-harvest processing, highlighting an imperative need to evaluate its microbial safety during pre-harvest practice. This study accessed the microbiological safety of blueberry produced in a commercial blueberry field applied with or without manure-derived ammonium sulfate (AS) fertilizer in a 2-year study. Indicator microorganisms of total coliforms and generic *E. coli*, Shiga toxin-producing *Escherichia coli* (STEC), *Salmonella*, and *Listeria monocytogenes* were monitored in fertilizer, soil, foliar, and blueberry fruit samples by culture methods for each production season. The population of total coliforms in soils was 3.17–3.82 Log_10_ CFU/g, which was stable throughout the production season and similar between two cropping seasons. Generic *E. coli* in soils remained at very low levels throughout the 2018 production season. Total coliforms or generic *E. coli* was not detected in fertilizer, foliar, and blueberry fruit samples collected in both 2017 and 2018 production seasons. STEC and *L. monocytogenes* were below the detection limit in fertilizer, soil, foliar, and blueberry fruit samples collected in both production seasons. *Salmonella* was not detected except for soil samples collected pre- and post-fertilizer application in the 2018 cropping season. Collectively, data indicated, under good agricultural practices, blueberry fruits produced in the field with or without manure-derived AS fertilizers had no microbiological safety concern.

## Introduction

The United States is the largest blueberry producer in the world, and Washington State is one of the leading blueberry producers in the United States (Evans and Ballen, 2014). Blueberries are grown in an open environment and subjected to potential contamination of foodborne pathogens from the environment including soil, irrigation water, wild animals, and runoff water from the surrounding cattle farms during production like other fruits (Doyle and Erickson, 2008). In addition, blueberries as ready-to-eat fruits usually have no or limited antimicrobial interventions during post-harvest processing/packing or before consumption. This is also true for blueberries that are individually quick frozen, which is the most common market avenue for northwestern Washington producers, as there is no kill step during processing. Any contamination during blueberry production and pre-harvest handling can pose a food safety threat to consumers. Blueberry is listed in the top 10 riskiest foods by the U.S. Food and Drug Administration (FDA) (CSPI, 2009). In fact, fresh blueberries were linked to a *Salmonella* Newport outbreak in 2010 (Miller et al., 2013). Fresh and frozen blueberries and other berry products have been involved in multiple recalls due to potential contamination of foodborne pathogens including Shiga toxin-producing *Escherichia coli* (STEC) (Luna and Mody, 2010), *Salmonella* (Larsen, 2018), *Listeria monocytogenes* (FDA, 1998), and viruses including norovirus and hepatitis A virus (FDA, 2019a,b). Controlling and minimizing pre-harvest contamination is considered as one of the key aspects of microbiological safety for blueberry production.

Raw or processed manures are commonly used as soil amendments for blueberry production (Benedict et al., 2018), and are cost-effective alternatives to synthetic fertilizers. Anaerobic digestion of manure reduces adverse impacts of raw manure on the environment such as odor and greenhouse gas emission (Holm-Nielsen et al., 2009) and reduces fecal coliform levels in the manure (Saunders et al., 2012). Ammonium sulfate (AS), the most frequently used type of nitrogen fertilizer for blueberry production (Vargas and Bryla, 2015), is either produced from chemically processed products or anaerobically digested manure (Benedict et al., 2018). Manure-derived AS captures ammonium and directly provides plant-available minerals for crops (Chien et al., 2011), and also overcomes the drawbacks of applying raw manure including the difficulties of transporting and storing and the concerns of affecting air, water, and soil qualities (Yorgey et al., 2014; Benedict et al., 2018). As a result, AS is commonly used in blueberry production, which improves berry yield (Rempel et al., 2004). Though anaerobic digestion of manure reduces pathogens (Yorgey et al., 2014), its efficacy of pathogen reduction varies depending on pathogen type and anaerobic digestion condition (Aitken et al., 2007; Masse et al., 2011). Thus, manure-derived AS could contain pathogens, and there is a need to evaluate the potential impact of its application on microbial safety of produced blueberry.

Blueberry production in northwestern Washington occurs in proximity to dairy production that further increases pathogen contamination risks. As a result, besides pathogen introduction from biological soil amendments, the runoff from dairy farms could increase the potential contamination risk of foodborne pathogens (Doyle and Erickson, 2008). This was highlighted by a recent *E. coli* O157:H7 outbreak linked to romaine lettuce (FDA, 2018b), where the runoff from adjacent dairy farm was identified as a potential source of *E. coli* O157:H7. In addition, soil could be contaminated by foodborne pathogens from irrigation water (Steele and Odumeru, 2004) and domestic/wild animal feces (Gorski et al., 2011). The contaminated soil could further contaminate the fresh produce through aerosols under harsh weather such as strong winds (Baertsch et al., 2007). Furthermore, worker hygiene and sanitation practices during blueberry production play a role in microbial contamination due to their contacts with both soil and crop (Gurtler, 2017). Therefore, examining the prevalence of foodborne pathogens in soil and fruits is critical to ensure pre-harvest food safety.

Currently, there is no information available regarding the potential risk of foodborne pathogen contamination during blueberry production in the field with or without manure-derived AS. By analyzing two blueberry production seasons (2017–2018), this study aimed to dynamically evaluate the pre-harvest microbiological safety of blueberry production by monitoring indicator microorganisms, total coliforms and generic *E. coli*, and the main foodborne pathogens including STEC, *Salmonella*, and *L. monocytogenes* in fertilizers, soil, foliar, and blueberry fruit samples.

## Materials and Methods

### Field Design and Sample Collection

The experiment was conducted in a 10-year-old 1.25-ha “Draper” blueberry field located in Whatcom County, which is the leading berry production area in Washington (DeVetter et al., 2015) as well as ranked the 38th in the United States for milk production (USDA, 2018). The farm implemented Good Agricultural Practices (GAP) during blueberry production (USDA, 1998). The irrigation water was filtered and treated with chlorine before use. The fences were installed, and the signage was used for exclusion of domestic animals. All workers took food safety training and had access to portable toilets equipped with hand washing stations and hand sanitizer.

**Figure 1:**
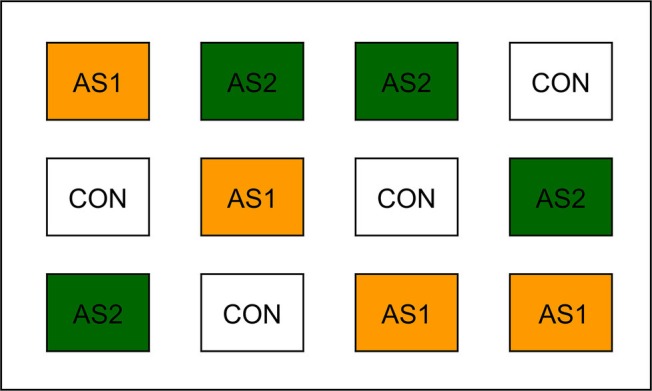
Fertilizer application on the designed plots in 2017–2018 production seasons. CON, standard fertilization; AS1, manure-derived ammonium sulfate; AS2, manure-derived ammonium sulfate applied in split applications.

The monthly temperature in March, April, May, June, July, and August during 2017–2018 production seasons was 6.4–7.3, 9.6–10.0, 13.1–14.2, 15.4–15.7, 17.5–18.8, and 17.6–18.1°C, respectively. The precipitation ranged from 0.00 to 0.48 cm, the wind speed ranged from 2.53 to 3.74 m/s, and the humidity ranged from 66.62 to 82.96% during the 2017–2018 production seasons (Weatherunderground, 2019). Individual blueberry plots measured 22.86 m × 3.05 m containing one row of blueberry plants irrigated with water using two buried drip lines. Standard dry synthetic fertilizer as control (CON) was supplied by CHS Northwest (Lynden, WA) and applied using a side-discharge fertilizer spreader. Liquid AS, a fertilizer derived from anaerobic digestion of raw manure straight lagoon (Benedict et al., 2018), was applied *via* CO_2_ backpack sprayer (Bellspray Inc., Opelousas, LA) at 32 pounds per square inch (PSI). Treatment plots were set up in a completely randomized design (Figure 1). AS was extracted from the anaerobically digested raw manure straight lagoon through ammonia stripping process (Benedict et al., 2018; Frear et al., 2018) by Regenis Corporation of Ferndale (Lynden, WA). AS was either applied once for each production season (AS1) or applied as a split application 4 weeks apart (AS2). The total amount of AS applied to each blueberry plot was the same. AS1 and the first portion of AS2 application occurred on the same day to deliver identical total nitrogen at the same rate as CON (based on commercial grower standard). AS was applied directly to soil at the base of the blueberry plants. Physiochemical properties including nutritional information and moisture content of AS and CON are summarized in Table 1. The pH of fertilizer samples is listed in Table 2.

**Table 1:** Physiochemical properties of tested fertilizers.

Fertilizer treatment	Moisture (%)	Nitrogen		Phosphorus (g P_2_O_5_/kg)	Potassium (g K_2_O/kg)
		NH_4_ (g/kg)	Total N (g/kg)		
CON	/	110.00	120.00	130.00	140.00
AS1	100.0	52.44	50.39	0.001	0.01
AS2	100.0	52.44	50.39	0.001	0.01

*Values were averaged from 12 samples/fertilizer over the 2-year study (6 samples/year, *n* = 12). /, not measured; CON, standard fertilization; AS1, manure-derived ammonium sulfate; AS2, manure-derived ammonium sulfate applied in split applications*.

**Table 2:** pH of fertilizer, soil, foliar, and blueberry fruit samples in 2017–2018.

Fertilizer treatment	Fertilizer	Soil	Foliar	Fruit
**CON**	/	4.69 ± 0.07	6.05 ± 0.01	3.51 ± 0.03
**AS1**	1.77 ± 0.00	4.65 ± 0.12		
**AS2**	1.77 ± 0.00	4.75 ± 0.10		

*Data are represented as mean ± SEM, *n* = 12, 6 and 32 for fertilizer, foliar or fruit and soil, respectively. The pH of soil was averaged from pH of soil samples collected at March (pre-fertilizer application), April (post-fertilizer application), June and August of each production season, four samples per sampling points, thus a total of 32 samples. /, not measured; CON, standard fertilization; AS1, manure-derived ammonium sulfate; AS2, manure-derived ammonium sulfate applied in split applications*.

**Figure 2:**
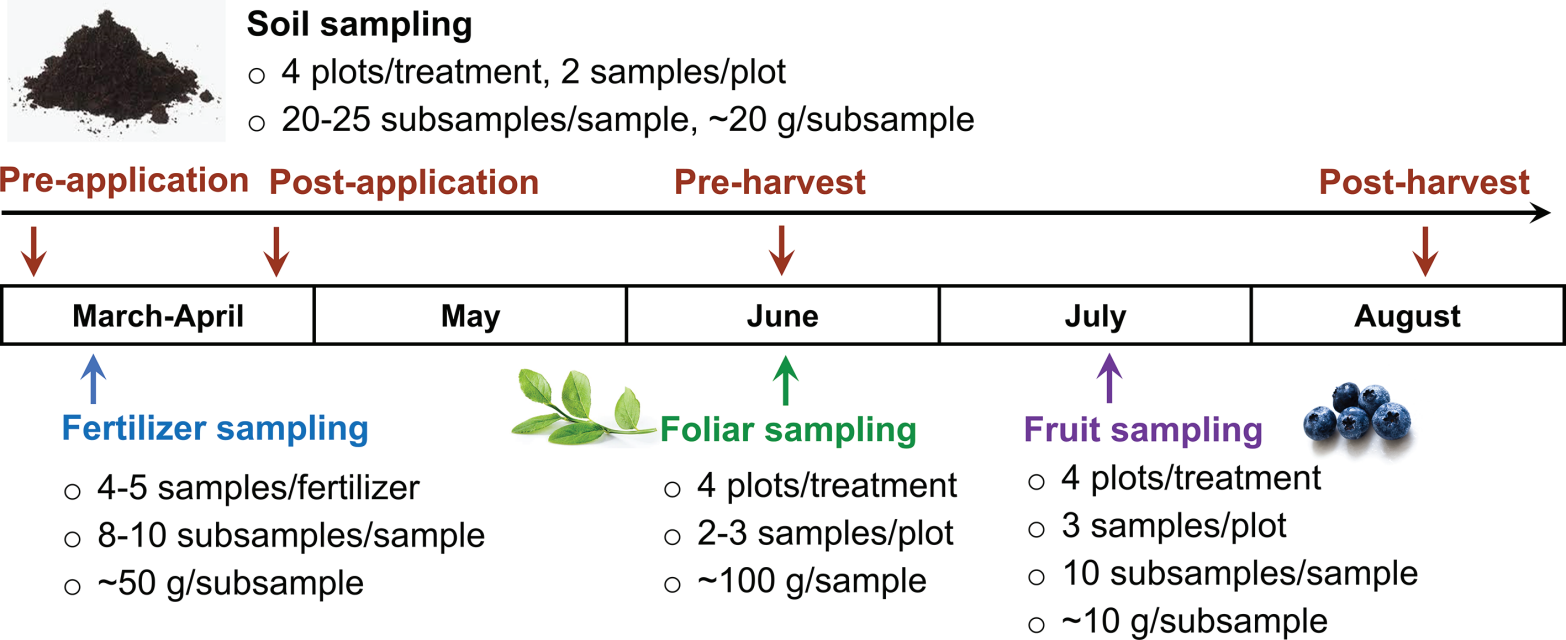
Microbiological sample collection plan in 2017–2018 production seasons.

The fertilizer products were sampled before applying to soils in late March. For CON fertilizer sampling, 8–10 subsamples (~50 g each) were taken from three depths, and placed in an one liter polyethylene bottle (Nalgene, Thermo Fisher Scientific Inc., Waltham, MA). AS1 and AS2 samples were taken from a 100-gallon liquid container, well mixed before sampling. Soil samples were collected pre- and post-fertilizer application, as well as pre- and post-fruit harvest for both 2017 and 2018 production seasons (Figure 2), because soil could be a pathway for introducing foodborne pathogens to crop from pre-harvest practices and environmental contaminations. Individual soil samples were collected at a depth of 0–5 cm in the plot by a soil core sampler (2.5 cm diameter) and placed into a Ziploc storage bag (Johnson & Son, Racine, WI) (EPA, 2014). Each soil sample (400–500 g) composing of 20–25 subsamples (~20 g/subsample) was collected in duplicate from each plot. Both foliar and blueberries were collected to investigate the microbiological safety of crop under current pre-harvest practices (Figure 2). Foliar samples (~100 g per sample) were randomly collected from the bushes at pre-harvest in duplicate per plot and placed in paper bags. Blueberries were collected in triplicate from each plot with ~100 g/sample and placed into a plastic clamshell container to avoid damage (Figure 2). All the collected samples were immediately chilled on ice in an insulated cooler and transported to the Washington State University Food Microbiology laboratory (Pullman, WA) for analysis within 24 h. The pH of soil, foliar, and blueberry fruit samples was further measured and is summarized in Table 2.

### Microbial Indicator Analysis

Generic *E. coli* was previously used as an indicator for pathogenic bacterial contamination in field studies (Natvig et al., 2002). Thus, generic *E. coli* along with total coliforms were analyzed as microbial indicators in this study. Microbial indicator analysis was conducted following FDA’s Bacteriological Analytical Manual (BAM) (FDA, 2002) with minor modifications. Total coliforms and generic *E. coli* of soil samples were enumerated by both directly plating and 3-tube most probable number (MPN), while only MPN quantification was used for fertilizer, foliar, and blueberry fruit samples. Each representative sample of 25 g was homogenized in 225-ml buffered peptone water [BPW, Becton, Dickinson and Company (BD), Sparks, MD]. The well-mixed bacterial suspension was serially diluted and plated onto duplicate plates of CHROMagar ECC (CHROMagar, Paris, FR), followed by incubation at 30 and 44.5°C for 24 h for enumeration of total coliforms and generic *E. coli*, respectively. For MPN determination, 1 ml of the above bacterial suspension was transferred to three tubes containing 9 ml of lauryl sulfate broth [LST, Hardy Diagnostics (HD), Santa Maria, CA] and incubated at 35°C for 24–48 h. Positive tubes that showed both turbidity and gas production were further confirmed in brilliant green lactose bile (BGLB, HD) broth and *E. coli* broth with 4-methylumbelliferyl-β-D-glucuronide (EC + MUG, HD) for total coliforms and generic *E. coli*, respectively. BGLB tubes were incubated at 35°C for 24–48 h, and tubes that showed turbidity and gas production were considered coliform positive. EC + MUG tubes were incubated at 44.5°C for 48 h, and tubes that showed turbidity, gas production and fluorescent under a long-wave UV lamp were considered generic *E. coli* positive.

### Pathogen Detection and Confirmation

#### Shiga Toxin-Producing *Escherichia coli*

Presumptive STEC were isolated following BAM method (Feng et al., 2011). Each representative sample of 25 g was homogenized in modified BPW with 0.1% (w/v) pyruvate (mBPWp, Amresco, Solon, OH), incubated at 35°C for 5 h, then supplemented with 10 mg/l acriflavine (TCI, Portland, OR), 10 mg/l cefsulodin (Sigma-Aldrich, St. Louis, MO), and 8 mg/l vancomycin (Sigma-Aldrich), mixed and incubated at 42°C for additional 18–24 h. The overnight enrichment cultures were serially diluted and plated onto CHROMagar STEC (CHROMagar) and confirmed by PCR assay (Feng and Monday, 2000). In addition, the overnight enrichment cultures were used to extract DNA and subject to multiplex PCR assay for the detection of STEC per the BAM method by targeting *uidA* with *stx1* and/or *stx2* for STEC per previously published methods (Feng et al., 2011).

#### Salmonella

The isolation of *Salmonella* spp. was conducted per the BAM method (FDA, 2011). Each representative sample of 25 g was homogenized in BPW and enriched at 35°C for 24 h. One milliliter of the overnight culture was sub-cultured to 10 ml of tetrathionate broth (TT, BD) supplemented with 2% iodine-iodide solution (HD) and incubated at 35°C for 24 h, or 0.1 ml of the overnight culture was sub-cultured to 10 ml of Rappaport-Vassiliadis broth (RV, BD) and incubated at 42°C for 24 h. The resulting enrichment culture was streaked onto xylose lysine deoxycholate (XLD, HD), bismuth sulfite (BS, HD), hektoen enteric (HE, HD) and CHROMagar *Salmonella* (CHROMagar) plates and incubated at 37°C for 24–48 h to discriminate *Salmonella*. Presumptive colonies were defined as the pink colonies with or without black centers on XLD, the brown, gray, or black colonies with or without metallic sheens on BS, the blue-green colonies with or without black centers on HE, and the mauve colonies on CHROMagar *Salmonella* plate. The presumptive colonies were further confirmed by *Salmonella* Latex Test (Oxoid Ltd., Hants, UK) and by PCR targeting *Salmonella* invasive gene *invA* (Rahn et al., 1992). *Salmonella* positive samples were further enumerated by the modified 3-tube MPN method (Fravalo et al., 2003) and interpreted according to BAM with the detection limit of 3 MPN/g (FDA, 2010). The enumeration test was performed a day after the confirmation of the presence of *Salmonella*.

#### Listeria monocytogenes

To isolate *L. monocytogenes*, 25 grams of respective samples were suspended in 225 ml of buffered *Listeria* enrichment broth (BLEB, BD), incubated for 4 h at 30°C, then supplemented with acriflavine (TCI), nalidixic acid (Sigma-Aldrich), and cycloheximide (Amresco) for an additional selective enrichment at 30°C for 24–48 h. The enrichment culture was streaked onto both Modified Oxford agar (MOX, BD) and CHROMagar *Listeria* (CHROMagar) plates and incubated at 37°C for 24–48 h. Presumptive colonies were defined as smooth, round, turquoise colonies 1–1.5 mm in diameter surrounded by an opaque white halo on MOX, and blue colonies with white halo on CHROMagar *Listeria* plates, and were confirmed by PCR targeting the different region of the invasion-associated secreted endopeptidase (*iap*) gene (FDA, 2017).

### Statistical Analysis

The total coliforms and generic *E. coli* in soil samples per treatment were analyzed with one-way analysis of variance (ANOVA) and least significant difference (LSD) multiple-comparison test using IBM SPSS 19.0 (Chicago, IL). Results were considered significant at *p* ≤ 0.05. Data were reported as mean ± SEM (standard error mean).

## Results

### Enumeration of Indicator Microorganism in Fertilizer, Soil, Foliar, and Blueberry Samples

**Figure 3:**
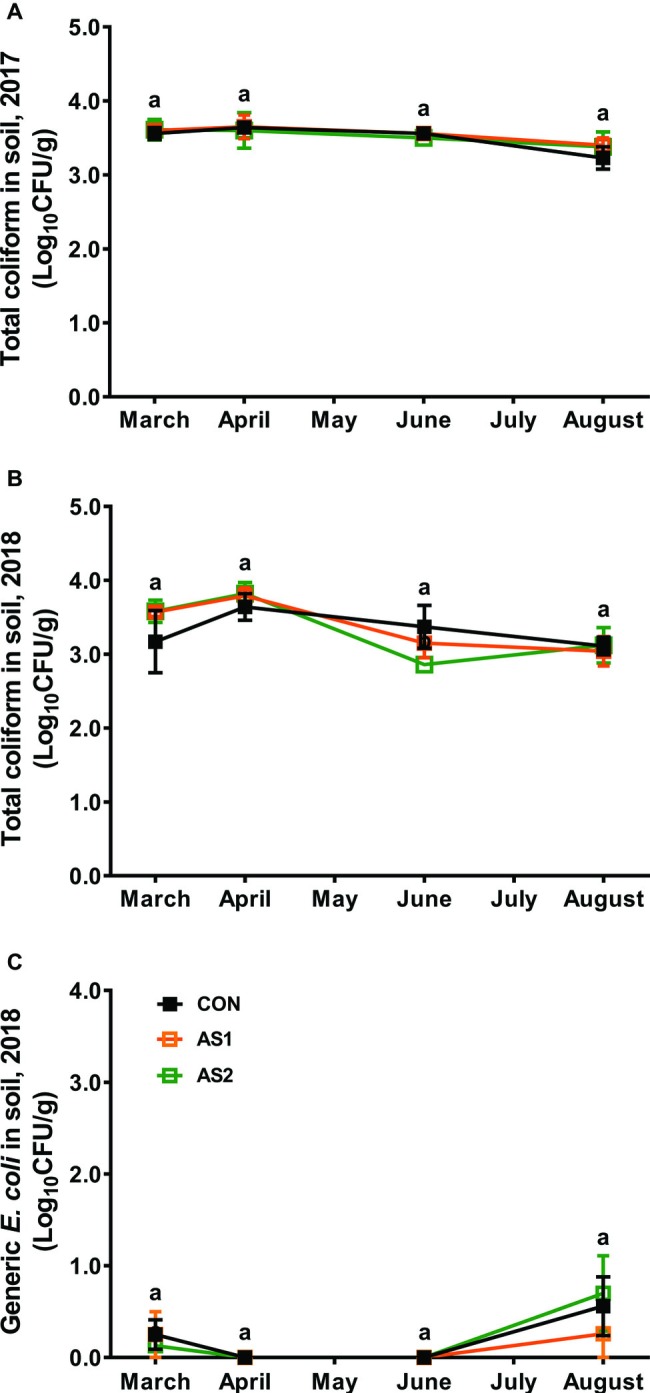
Abundance of total coliforms in soil samples collected from the blueberry field during 2017–2018 production seasons. **(A)** Total coliform 2017; **(B)** Total coliform 2018; **(C)** Generic *E. coli* 2018. *n* = 4. Means at each sampling point without common letter differ significantly (*p* < 0.05). CON, standard fertilization; AS1, manure-derived ammonium sulfate; AS2, manure-derived ammonium sulfate applied in split applications.

Total coliforms and generic *E. coli* were analyzed to represent the fecal contamination potential of the tested samples (Lonigro et al., 2016). In both 2017 and 2018 production seasons, total coliforms or generic *E. coli* were not detected in any fertilizer, foliar, and blueberry fruit samples by the MPN method with the detection limit of 3 MPN/g (data not shown). The population of total coliforms in soils was ~3.5 Log_10_ CFU/g and relatively stable throughout the 2017 production season regardless of fertilizer application (Figure 3A). Similar phenomenon was observed in the 2018 production season (Figure 3B). Generic *E. coli* stayed at very low levels of 0.13–0.25 Log_10_ CFU/g throughout the 2018 production season regardless of fertilizer application/practices (Figure 3C).

### Detection of Pathogens in Fertilizer, Soil, Foliar, and Blueberry Samples

STEC were not detected in fertilizer, soil, foliar, or blueberry fruit samples throughout 2017–2018 production seasons either by multiplex PCR or direct plating method followed by PCR confirmation of all the presumptive STEC colonies (1–10 colonies per plate, targeting Shiga toxin genes *stx1* and *stx2* with the detection limit of 1 CFU/g) (Table 3; Feng and Monday, 2000). In 2017, *Salmonella* were not detected in fertilizer and soil samples collected at all sampling stages throughout the production season, nor were they detected in foliar or blueberry fruit samples regardless of fertilizer application (Table 4). In the 2018 cropping season, *Salmonella* were not detected in fertilizer samples but were detected in all soil samples collected pre-fertilizer application and some of those collected post-fertilizer application (Table 4). The level of *Salmonella* contamination ranged from 0.48 to 1.04 Log_10_ MPN/g in soil (Table 5). *Salmonella* were not detected in soil samples collected before or after fruit harvest, nor were they detected in foliar or blueberry fruit samples (Table 4). In both production seasons, *L. monocytogenes* were not detected in fertilizer, soil, foliar, or blueberry fruit samples regardless of fertilizer application practices (Table 6).

**Table 3:** STEC analyses in fertilizer, soil, foliar, and blueberry fruit samples in 2017–2018.

Treatment	Fertilizer	Soil				Foliar	Fruit
		March–April		June	August		
		Pre	Post				
**CON**	0/5^a^	0/4	0/4	0/4	0/4	0/4	0/4
**AS1**	0/5	0/4	0/4	0/4	0/4	0/4	0/4
**AS2**	0/5	0/4	0/4	0/4	0/4	0/4	0/4

a *Positive samples/total samples for each treatment. 2017 and 2018 production seasons with the same result was only reported once. Shiga toxin-producing E. coli (STEC) was detected by CHROMagar STEC plates and confirmed by PCR or multiplex PCR assay. CON, standard fertilization; AS1, ammonium sulfate; AS2, ammonium sulfate applied in split applications; Pre/Post, soils were sampled pre-application/post-application of fertilizers in late March and early April; June, soils were sampled in June before fruit harvest; August, soils were sampled in August after fruit harvest*.

**Table 4:** Detection of *Salmonella* in fertilizer, soil, foliar, and blueberry fruit samples in 2017–2018.

Treatment	Fertilizer	Soil				Foliar	Fruit
		March–April		June	August		
		Pre	Post				
**CON**	0/4^a^	0/8**|8/8^b^**	0/8**|1/8**	0/8	0/8	0/12	0/12
**AS1**	0/4	0/8**|8/8**	0/8**|2/8**	0/8	0/8	0/12	0/12
**AS2**	0/4	0/8**|8/8**	0/8**|2/8**	0/8	0/8	0/12	0/12

a *Positive samples/total samples for each treatment; 2017 and 2018 production seasons with the same result was only reported once*.

b *Positive samples/total samples in 2017|Positive samples/total samples in 2018; the positive results were confirmed by Latex Salmonella and PCR detection of invA gene; CON, standard fertilization; AS1, manure-derived ammonium sulfate; AS2, manure-derived ammonium sulfate applied in split applications; Pre/Post, soils were sampled pre-application/post-application of fertilizers in late March and early April; June, soils were sampled in June before fruit harvest; August, soils were sampled in August after fruit harvest*.

**Table 5:** Most probable number determination of *Salmonella* in soil samples collected during 2018 production season.

Treatment	March–April		June	August
	Pre	Post		
**CON**	<1.04 ± 0.37^a^	<0.52 ± 0.05	/	/
**AS1**	<0.48 ± 0.00	<0.48 ± 0.00	/	/
**AS2**	<0.67 ± 0.13	<0.49 ± 0.01	/	/

a *Most probable number, Log_10_ MPN/g, Mean ± SEM, n = 8; /, not enumerated; CON, standard fertilization; AS1, manure-derived ammonium sulfate; AS2, manure-derived ammonium sulfate applied in split applications; Pre/Post, soils were sampled pre-application/post-application of fertilizers in late March and early April; June, soils were sampled in June before fruit harvest; August, soils were sampled in August after fruit harvest*.

**Table 6:** *Listeria monocytogenes* analyses in fertilizer, soil, foliar, and blueberry fruit samples in 2017–2018.

Treatment	Fertilizer	Soil				Foliar	Fruit
		March–April		June	August		
		Pre	Post				
CON	0/5^a^	0/4	0/4	0/4	0/4	0/4	0/4
AS1	0/5	0/4	0/4	0/4	0/4	0/4	0/4
AS2	0/5	0/4	0/4	0/4	0/4	0/4	0/4

a *Positive samples/total samples for each treatment. L. monocytogenes was detected by both Modified Oxford agar and CHROMagar Listeria plates. 2017 and 2018 production seasons with the same result was only reported once. CON, standard fertilization; AS1, ammonium sulfate; AS2, ammonium sulfate applied in split applications; Pre/Post, soils were sampled pre-application/post-application of fertilizers in late March and early April; June, soils were sampled in June before fruit harvest; August, soils were sampled in August after fruit harvest*.

## Discussion

Fresh produce is recently recognized as a main foodborne source for STEC, *Salmonella*, and *L. monocytogenes* (Luna and Mody, 2010; CDC, 2012; Miller et al., 2013). Cattle are the main reservoir of STEC, *Salmonella*, and *L. monocytogenes* (Heredia and Garcia, 2018). STEC can be shed in manure, transferred to soil, and persist for an extended period (Fremaux et al., 2008). The manure-derived AS might carry pathogens originated from gastrointestinal tract of healthy animals (Aitken et al., 2007). Once applied, manure-derived fertilizers further transfer pathogens to fresh produce, resulting in deadly outbreaks (CDC, 2018b). AS application does not require an interval time by the Produce Safety Rule under the Food Safety Modernization Act (FSMA) (FDA, 2018a), which might increase the chance of contamination of produce. Up to now, little is known about the microbial safety of blueberry produced with manure-derived AS application. We found that AS derived from anaerobic digestion of raw dairy manure was absent of indicator and pathogenic microorganisms, indicating no safety concern of using manure-derived AS for soil amendment in blueberry field. Split application of nitrogen fertilizer in blueberry production is a common agricultural practice, which results in higher yields than that of single application (Hanson and Retamales, 1992; Rempel et al., 2004). Our data indicated that the application method of manure-derived AS, single or split application, had no effect on the microbial safety of blueberry production.

Total coliforms and generic *E. coli* were previously used as hygiene indicators in soil and fresh produce (Natvig et al., 2002; Denis et al., 2016; Lonigro et al., 2016). Total coliform level in soils from the blueberry field remained relatively stable over seasons regardless of fertilizer application/practice, which was lower than that of soils of a parallel raspberry study (Sheng et al., 2019). This could probably be due to different production environment and/or agriculture practices. Generic *E. coli* remained at very low counts throughout the 2018 production season regardless of fertilizer application, which was consistent with a recent study in soils from a raspberry field (Sheng et al., 2019). Similarly, application of compost did not affect *E. coli* abundance in soils (Larney et al., 2003). Amending soils with anaerobically digested dairy manure did not affect the die-off rate of *E. coli* in soils (Saunders et al., 2012). Generic *E. coli* levels of post-fruit harvest soil samples were slightly increased, which was possibly due to the increased human activities and/or the environmental temperature in the late production season. Consistently, the transient residence of generic *E. coli* in soils was reported to be related to soil moisture and environmental temperature (Lang et al., 2007). In this study, total coliforms or generic *E. coli* were not associated with the presence of *Salmonella* in soils, suggesting they might not be appropriate indicator microorganisms in a berry production environment. Similar to our findings, the total coliform level of soil was not correlated with *Salmonella* presented in soils cultivating raspberry (Sheng et al., 2019) or lettuce (Holvoet et al., 2014). Generic *E. coli* was not associated with the presence of *Salmonella* in raspberry (Sheng et al., 2019) or leafy green produce field (Benjamin et al., 2013).

*E. coli* O157: H7 and non-O157 STEC have increasingly been implicated in deadly fresh produce outbreaks (CDC, 2017, 2018b). STEC were not detected in soil samples with different fertilizer applications using both traditional culture-based method and multiplex PCR method, indicating that blueberry production using manure-derived AS fertilizer did not pose a STEC risk. This was consistent with a recent study on raspberry field, where STEC was not detected in soil amended with various manure-based fertilizers (Sheng et al., 2019). Anaerobic digestion facilitates *E. coli* O157:H7 inactivation in dairy manure, especially at a high-temperature condition (Aitken et al., 2007). The acidic soil pH of blueberry fields might also contribute to inactivation of STEC, because the survival of STEC in soils was impaired by the acidic environment (Erickson et al., 2014).

*Salmonella* is stable in a dry environment and subsequent thermal treatment (Tsai et al., 2019) and develops resistance to a wide range of antibiotics (Sobsey et al., 2006). *Salmonella* has frequently been involved in fresh produce outbreaks (Miller et al., 2013; CDC, 2018a). In the 2018 cropping season, *Salmonella* was detected in early season soil samples regardless of fertilizer application. Similarly, *Salmonella* were not detected in anaerobically digested manure but were isolated from soils after anaerobically digested manure application (Goberna et al., 2011; Sheng et al., 2019). The 2.6 and 2.2% of soil samples collected from produce fields in California and New York State, respectively, were positive of *Salmonella* (Gorski et al., 2011; Strawn et al., 2013). The detection of *Salmonella* in blueberry field could be due to precipitation (Gorski et al., 2011), wild animal feces (Gorski et al., 2011), irrigation water (Steele and Odumeru, 2004), farm animal activity (Nottingham and Urselmann, 1961), and others. The precipitation in Whatcom County, Washington, was higher during pre- and post-fertilizer application in March–April 2018 compared to that in late production seasons, which might contribute to the transient presence of *Salmonella* in early season soil samples. The exact reason for the reduced prevalence of *Salmonella* in post-fertilizer soil samples was unknown. However, this could be due to additional die-off with time and the interaction of *Salmonella* with fertilizers and soils. Indeed, soil temperature, pH and moisture content as well as soil microbial community could affect the survival of *Salmonella* in soils and result in the dynamic change of *Salmonella* (Bradford et al., 2013). To maximize the recovery and detection of *Salmonella,* different selective media were used in this study, due to the different performance of the existing selected selective media (Pal and Marshall, 2009).

*L. monocytogenes* poses a unique challenge to fresh produce due to its high mortality rate and ubiquitous nature in environment (Weis and Seeliger, 1975; CDC, 2012). *L. monocytogenes* are commonly isolated from the feces of dairy cattle (Heredia and Garcia, 2018) and can survive in manure for up to 6 months (Nicholson et al., 2005). In this study, *L. monocytogenes* were not detected in soil samples amended with CON or AS. In support of our finding, *L. monocytogenes* were not detected in soils that were amended with various manure-based fertilizers culturing raspberry (Sheng et al., 2019) or leafy green (Rahube et al., 2014).

Consistent with soil microbial results, neither indicator microorganisms nor pathogenic bacteria were detected from foliar or blueberry fruit samples collected during both cropping seasons regardless of fertilizer treatments. Similar to our findings, pathogenic bacteria or generic *E. coli* were not detected in ~500 blueberries samples according to a 4-year survey (Denis et al., 2016). The most common type of blueberry cultivated in Washington State are highbush blueberries (*Vaccinium corymbosum*) at 6–10 feet tall (Strik et al., 2014), where blueberries are less likely to directly contact with soils at growing season compared to lowbush blueberries. As a result, blueberry fruits are less likely to be contaminated during production compared to produce such as lettuce grown close to soil (Murphy et al., 2016). In addition, the low pH of blueberry fruits might provide another hurdle against foodborne pathogens (Nguyen et al., 2014).

## Conclusion

This 2-year field study demonstrated that, under good agricultural practices, blueberries produced in fields with or without manure-derived fertilizer AS had little food safety concern. STEC and *L. monocytogenes* were all below the detection limit in all samples collected during both production seasons. *Salmonella* were below the detection limit in all samples except early season soil samples collected in 2018 production season. Additionally, the total coliforms in soil samples remained stable throughout the production seasons and were not detectable in fruit samples. This study provides valuable information for the blueberry industry regarding microbiological safety concern of close proximity to dairy production and the use of manure-derived fertilizers.

## Data Availability Statement

All datasets generated for this study are included in the article.

## Author Contributions

XS, LS, and YS performed the experiment. M-JZ, CK, and CB designed the experiment. XS and M-JZ wrote the manuscript. CB and ES helped with sample collection. M-JZ, CB, CK, LS, and YZ revised the manuscript.

### Conflict of Interest

The authors declare that the research was conducted in the absence of any commercial or financial relationships that could be construed as a potential conflict of interest.
